# Dynamic Tire Pressure Sensor for Measuring Ground Vibration

**DOI:** 10.3390/s121115192

**Published:** 2012-11-07

**Authors:** Qi Wang, James Gregory McDaniel, Ming L. Wang

**Affiliations:** 1 Department of Electrical and Computer Engineering, Northeastern University, Boston, MA 02115, USA; 2 Department of Mechanical Engineering, Boston University, Boston, MA 02215, USA; E-Mail: jgm@bu.edu; 3 Department of Civil and Environmental Engineering, Northeastern University, Boston, MA 02115, USA

**Keywords:** non-contact, instantaneous, dynamic tire pressure sensor, ground acceleration, pavement condition

## Abstract

This work presents a convenient and non-contact acoustic sensing approach for measuring ground vibration. This approach, which uses an instantaneous dynamic tire pressure sensor (DTPS), possesses the capability to replace the accelerometer or directional microphone currently being used for inspecting pavement conditions. By measuring dynamic pressure changes inside the tire, ground vibration can be amplified and isolated from environmental noise. In this work, verifications of the DTPS concept of sensing inside the tire have been carried out. In addition, comparisons between a DTPS, ground-mounted accelerometer, and directional microphone are made. A data analysis algorithm has been developed and optimized to reconstruct ground acceleration from DTPS data. Numerical and experimental studies of this DTPS reveal a strong potential for measuring ground vibration caused by a moving vehicle. A calibration of transfer function between dynamic tire pressure change and ground acceleration may be needed for different tire system or for more accurate application.

## 1. Introduction

Various sensor systems are widely used in seismic testing and pavement inspection [1,2]. Accurate, economic, and efficient non-destructive testing techniques are highly desired for evaluation of seismic data for infrastructure integrity. Some alternative ways of using acoustic microphones, accelerometers and geophones have been investigated to meet the increasing demands and to prevent the major infrastructure from undergoing irreversible changes [3–5]. It has been widely recognized that the propagation of surface acoustic waves through concrete and asphalt layers can be measured by accelerometers or microphones; the propagation characteristics can be used to estimate the material and structural properties, including the existence of damage on the surface and in the sub-surface of the structure [1–6].

Static systems relying upon an external impact source to produce acoustic signals measureable with an acoustic transducer or an array of acoustic transducers are considered an effective way for any seismic tests, surface and subsurface sensing [2]. One acoustic wave-based method for detecting the seismic data and subsurface pavement profile is the air-coupled surface wave measurement using multichannel analysis of surface wave (MASW) technique [3,4]. A microphone array is suspended in the air, close to the ground, to collect leaky surface waves excited by a nearby hammer impact. The typical test setup with two receivers of spectral analysis of surface waves (SASW). The receivers usually used in MASW are velocity transducers (geophones) and acceleration transducers (accelerometers). After the entire test is completed, an iterative dispersion analysis, relying upon manual experience and intervention, is executed in order to achieve the estimation of subsurface profile. However, the data analysis is sensitive to the interference of ambient noise and hammer noise. The limit of lower frequencies which corresponds to the interrogation deeper layers is limited by the natural frequency of the receivers as well as frequencies being generated by the source. In the time domain, the surface wave can be separated from the direct hammer noise in subsequent data processing. The manipulation from test point to test point is rather slow. Besides, an external impact excitation is not capable of measuring the surface condition.

There is a significant need for a time-efficient inspection system with an automatic excitation source. Tire and road surface interactions produce acoustic signals, and these signals are measureable with an acoustic transducer or an array of acoustic transducers [7–9]. Specifically, using the tire as a mechanical excitation source eliminates the need for external impact used in previous sensor systems. Prior methods and systems using directional microphones needs to be placed close to the tire and suspended close to the ground on a moving cart or vehicle [10–12]. Aside from the obvious risk of transducer impact with the ground, there is a risk that the ambient noise would prevent the detection of acoustic signals from the tire-road interface and surface waves that propagate in the road. Moreover, for most cases, sophisticated signal processing methods are typically required in such analysis.

Roadway surface vibrations, circumferential tire vibrations and acoustic waves are generated as the tire rolls over the surface of asphalt, concrete or other roadway surface. The vibrations travel as surface waves on the road and through the rubber of the tire and radiate acoustic waves in the air. Acoustic waves depend on the material and structural properties of the volume through which they propagate; by the accurate recording of acoustic wave signals and necessary signal processing, one can characterize the structural and material properties of a surface. Therefore, an effective way to capture those tire excited acoustic waves with higher signal to noise ratio is vital to the design of the new system. Our novel approach is to place the sensor inside the tire and a dynamic tire pressure sensor is a perfect candidate for this requirement because it is noncontact [13–15].

The current method for monitoring the static tire pressure and its change over time is the tire-pressure monitoring system (TPMS) deployed in many modern cars [5]. This system was originally designed to identify under-inflation in any of the four tires of the vehicle. With a TPMS, static pressure sensors are mounted inside each tire to measure the static pressure every 30 seconds, and the information is wirelessly transmitted to the vehicle's instrument cluster. The sole purpose of the TPMS is to obtain the tire pressure and provide a low-pressure warning to the vehicle; therefore it does not provide a high sampling rate for tire pressure change, high transmitting rate, or an indication of dynamic tire pressure. In contrast, the proposed approach places a dynamic tire pressure sensor inside the tire. Specifically, a real-time dynamic tire pressure sensor (DTPS) system with a resolution of 0.02 milli-psi and low frequency response from 3 Hz has been attached to the tire though its valve stem. New hardware components and supporting signal processing strategies have also been developed. Other “intelligent tire” [16,17] or “smart tire” [18] systems use strain sensors [19,20]. These works focus on understanding wheel/ground friction by using strain sensors. Tire rubber deformation is not directly related to surface wave, ground vibration or road roughness. Often, complicated models are required in the interpretation between the PVDF strain sensor reading and wheel/ground friction. The purpose of friction measurement in these works is a safety concern of moving vehicle. Moreover, none of these strain sensors have been demonstrated on an actual moving tire with different road conditions where the temperature will be a major concern for these sensor's accuracy and lifetime.

The DTPS offers many advantages over previous approaches [1–5], such as measurement of a surface wave using several fixed accelerometers or several fixed directional microphones. First, the dynamic pressure is measured instead of static pressure. Testing can be performed while the vehicle is moving, as opposed to fixed testing. An instant/real-time pavement condition report is generated instead of an in-office, post-test report. The instrumented tire allows for fast, continuous testing, as opposed to slow testing due to frequent sensor mounting and removal; and it is suitable for thorough inspection of an entire pavement length. Depending upon data transmission requirements, the DTPS system can be either wired or wireless [21].

The present work focuses only on the feasibility of reconstructing ground vibration from DTPS measurement, and its ability to amplify ground vibration as compared to other sensors. This sensing approach allows measurements of ground vibration while the vehicle is moving, thus replacing stationary sensors that must be frequently repositioned and reattached. Since this is a completely novel application of DTPS with very few reported works in the open literature, many other fundamental investigations of DTPS have been done that are reported elsewhere, including comparison of DTPS and directional microphone for noise cancellation [22], effect of road profile on dynamic response of a vehicle using DTPS [23], real time wireless DTPS and its supporting energy harvesting system [24,25], and feasibility of DTPS in pavement assessment [25]. In this work, verification of the DTPS concept of sensing inside the tire and comparisons between DTPS, ground-mounted accelerometer and directional microphone have been carried out. A data analysis algorithm has been developed and optimized to reconstruct ground acceleration from DTPS data. Numerical and experimental studies of ground vibration have been conducted.

## 2. Theory and Method

**Figure 1.**
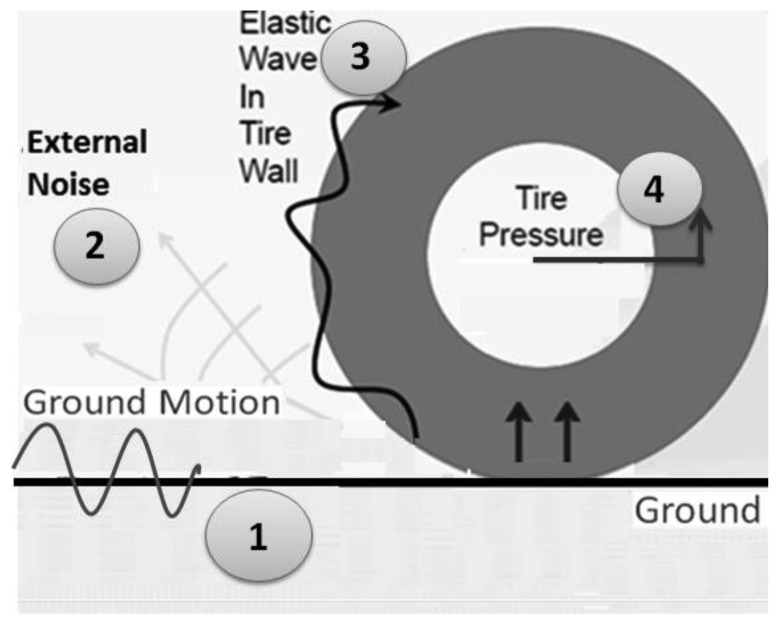
Mechanism of tire/road interaction and different acoustic sources.

Acoustic waves and ground vibration are generated while the vehicle is traveling on different road surfaces. They depend on the material properties of the road and vehicle. One of the most important issues for measuring ground vibration is to identify acoustic sources between the vehicle chassis and the road, which include vehicle body vibration, ground vibration, direct radiation from the tire-road interface and ambient. Figure 1 shows the mechanism of tire and road interaction. There are four distinct wave types that can be coupled to form acoustic waves due to complex interactions of tire dynamic with the road surface. These different effects on the tire are: (1) direct surface wave from impact source or vehicle; (2) acoustic radiation from vehicle and ambient; (3) elastic wave from this tire; (4) axle vibration [11–13]. The unique features of DTPS include: (1) the tire acts as a natural barrier to external noise; (2) the tire is directly in contact with the ground; (3) the instruments are protected from the environment; and (4) samples are taken in real-time.

**Figure 2.**
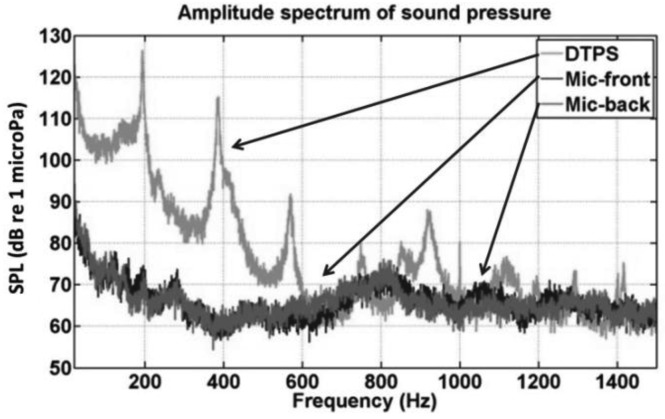
Comparison of SNR between DTPS and directional microphones.

Another important issue is the natural frequency of the acoustic resonance of the tire cavity. The cavity resonance is defined by the tire and rim size as well as the speed of sound in the acoustic medium that inflates the tire [7,16]. An approximate equation for the first natural frequency derived by finding the frequency, at which one acoustic wavelength would equal the average circumference of the tire:

(1) $$f=\frac{c}{l}=\frac{2c}{\pi (D+d)}$$

where *c* is speed of sound in the gas inflating the tire, *l* is average circumference of the tire cavity, *D* is the outer diameter of the cavity and d is inner diameter of the cavity. The tire size of the testing vehicle is LT245/75R16 with *D* of 30.7 inch and *d* of 16 inch, so that the cavity resonance is estimated at 190 Hz. This resonance was apparent in our previous work on a Signal to Noise Ratio (SNR) comparison between a DTPS and directional microphone [17–19]. One result from that work is shown in Figure 2. In this figure, the sound pressure level (SPL) is plotted versus frequency for driving on random highway with speed of 60 mph. Dynamic pressure inside the tire was measured by the DTPS while two directional microphones, in front of the tire and one in back, measure the acoustic pressure outside the tire. The first resonance peak near 200 Hz is very close to the estimate value above. More importantly, however, note that over the range of 0–600 Hz range, the SNR of the DTPS is approximately ten times (20 dB) higher than that either of the directional microphones.

Knowing the tire cavity resonance frequency is close to 195 Hz and its harmonics will be helpful for locating the featured ground motion characteristics in data analysis; however, these peeks can also be dominate for the useful signal in the same frequency ranges. Therefore, hardware or data analysis method of reducing tire resonance frequencies is needed for the DTPS application of measuring ground acceleration.

The use of the DTPS to detect surface and subsurface features of a roadway is similar to an acoustic impedance measurement in a multi-layered fluid system, which follows the following equation:

(2) $$Z=\frac{P}{\upsilon }$$

where P is the applied pressure at the point of contact between the road and the tire, or approximately 1/4 times the vehicle loading (N) divided by the tire to road contact area (Pa), and ν is velocity at the footprint of the tire [1]. Thus, the impedance Z contains information about subsurface properties and the vertical velocity v is directly related to the ground acceleration. It is important to establish a relationship between dynamic tire pressure change and the ground acceleration [14–16].

According to Boyle's Law, *PV^γ^* = *k*, where *P* is pressure, *V* is volume, *k* is a constant, and *γ* is specific heat (equals to 1.4 in air). Note this equation is valid under pure adiabatic condition which assumes the temperature of the air in the tire does not change. Thus, at any moment *t*, static tire pressure is *P* with the tire cavity volume of *V*.

For *t* = 0, 1, 2…m:

(3) $$\frac{P_{0}+\Delta P}{P_{0}}={\left(\frac{V_{0}}{V_{0}+\Delta V}\right)}^{\gamma }$$

where Δ*P* is the measured dynamic tire pressure sensor at any time, *P_0_* is the static tire pressure of the tire and *V_0_* is the volume of tire cavity at time *t* = 0 or without any impact input to the tire system. The volume change of the tire cavity is Δ*V* = *Ad*, where *A* is the tire footprint area on the road surface and d is the vertical displacement due to ground acceleration. This displacement may be found by integrating the measured acceleration twice with respect to time, so that one can compute ground acceleration *A_g_* that caused of dynamic tire pressure *P_m_*. Note the footprint area A of the testing van of the 2012 Chevrolet cargo van 3500 is estimated close to 31.2 in^2^, with the total vehicle loading of 9600 lbs. and tire pressure of 77 Psi.

## 3. Experimental Procedures

### 3.1. DTPS as Ground Vibration/Acceleration Amplifier

**Figure 3.**
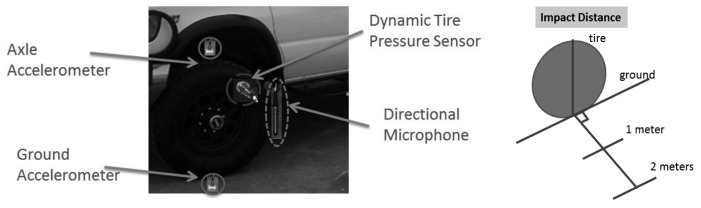
Test setup of DTPS as ground vibration amplifier validation.

In order to further assess the capability of DTPS as a ground vibration/acceleration amplifier, four sensors were used with the test van. A Chevrolet Express 3500 cargo van with tire model LT245/75R16E is used as the testing platform. A stationary test is performed with an external impact source (Kistler Force Hammer, ¼ lb., 1 lb. and 4 lbs.) at a distance of one and two meters from the tire/road surface contact point. Figure 3 illustrates the test configuration. A data acquisition unit (Data Physics Quattro), connected to a signal processing computer, is used for the four sensors, which are: (1) an accelerometer (Bruel & Kjaer Accelerometer 4507B004) mounted on the ground one inch from tire/road contact point; (2) a directional microphone (G.R.A.S. Directional microphone 40AE) suspended in the air one inch away from valve stem of the tire; (3) the DTPS (ICP pressure sensor 106B52) connected to the valve stem including a DTPS adapter fabricated to enable tire inflation up to 75 Psi without damaging the DTPS; and (4) an accelerometer (Bruel & Kjaer Accelerometer 4507B004) mounted on the axle of the test tire.

**Figure 4.**
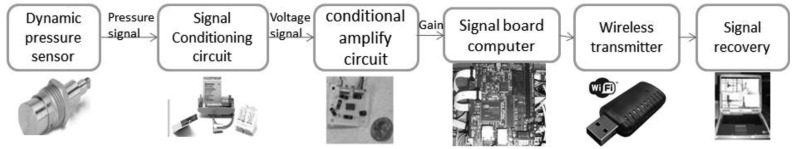
Wireless real-time DTPS system.

Note in a complete experimental configuration of DTPS system, the DTPS is installed on the valve stem of the rear tire of the cargo van. The wireless DTPS system, comprised of signal conditioning unit, amplifier, single board computer, and transmitter, was assembled and disposed in the center cap or hub cover of the respective wheel. A receiver is equipped together with a laptop inside the van. This will allow sampling rate up to 60,000 Hz. Building blocks of the system are shown in Figure 4.

### 3.2. DTPS Setup with Acoustic Damping Treatment inside Tire

**Figure 5.**
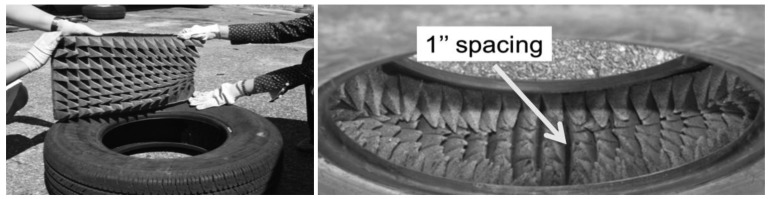
Tire with acoustic damping treatment.

In order to further improve the pure response of the DTPS to surface vibration, it was determined that acoustic response of the air in the tire at the cavity resonance should be decreased. In this way, the Signal to Noise Ratio of DTPS can be increased at those response resonance frequencies. One approach to achieving this goal is through the installation of acoustic damping material, such as acoustic foam sheets with pyramidal projections; glued into the tire the housed the DTPS. One inch spacing, shown in the right figure of Figure 5, is left on the inside of tire wall at the DTPS sensing point. Figure 4 shows the system setup for the acoustically treated tire.

### 3.3. DTPS Representing/Reconstructing Ground Acceleration

In order to calculate ground acceleration from measured DTPS data, same test setup mentioned in Section 3.1 and the acoustically damped tire configuration shown in Figure 5 are used.

### 3.4. Further Tests of DTPS Compared to Ground Vibration

Another test of the same setup as shown in Figure 5 in Section 3.2 is also performed, but input source is provided by the testing vehicle or axle of the testing tire instead of the hammer. This is achieved by jacking up car and release the testing tire (low frequency impact); and also by putting a generator (Honda EU2000i) inside the test van closer to the test tire (high frequency impact).

## 4. Results and Analysis

### 4.1. DTPS as Ground Vibration/Acceleration Amplifier

The test is conducted to determine whether DTPS acts as a ground vibration amplifier by sensing dynamic pressure inside the tire in order to measure the ground vibration caused by the passage of surface wave, to find out the best working frequency range of the DTPS, and to understand the events that cause the major contribution to the tire pressure change. Note that in our initial experiment of DTPS noise reveals that tire wall blocks more than 90% of the environmental noise, especially in the region from 0–3000 Hz. Meanwhile, DTPS as ground vibration amplifier is meaning the DTPS signal is higher than that of the suspended microphones. Moreover, the results from the DTPS with less than 1 Pa change also suggested that the air vibration excitation is not the only reason to cause major dynamic tire pressure changes.

**Figure 6.**
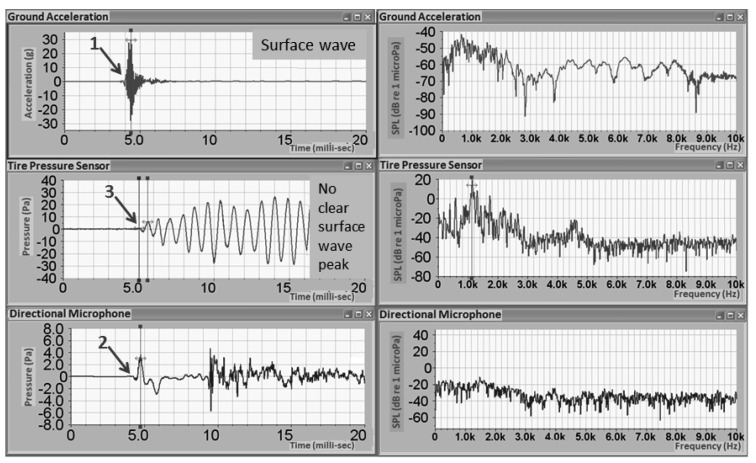
Results of hammer impact two meters away.

In this analysis, the test configuration shown in Figure 3 was used, along with a 1 lb. hammer strike approximately at two meter away from the tire. Figure 6 shows measurements from three in the time and frequency domains. Time domain results of a ground accelerometer, DTPS, and directional microphone are found on the left side of Figure 6. These three channels share the same starting time. The arrows mark the arrival time of the hammer impact to the sensors. Since the velocity of the surface wave in the pavement is around 1000 m/s, which is approximately three times that of the sound wave, the ground accelerometer shows the first arrival of the surface wave, which contributes most to the ground accelerometer. In the bottom left image, surface wave arrives at directional microphone afterward.

### 4.2. DTPS Setup with Foam

**Figure 7.**
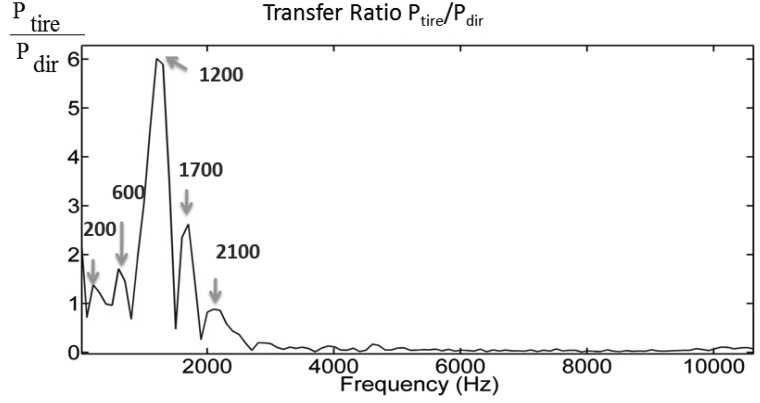
Transfer function of DTPS and directional microphone by the same hammer impact.

The results show a clear separation in the time domain between the surface wave and the acoustic wave from the same hammer impact. In the middle image (vertically), the same wave/excitation arrives at DTPS around 1ms later. There is no obvious separation between surface wave and acoustic wave and the tire pressure change is dominated by the cavity resonance in the frequency range from 50–1000 Hz. However, in the frequency analysis as shown on the right side of Figure 6, results from DTPS have a high signal to noise ratio over the other two sensors in the range near 1000 Hz, which could be related to the cavity resonance of the tire system. The transfer function, defined as the ratio of pressure amplitude inside the tire to the pressure amplitude outside the tire, is displayed in Figure 7. Compared to the directional microphone, DTPS amplifies the pressure in the region from 0–3000 Hz.

**Figure 8.**
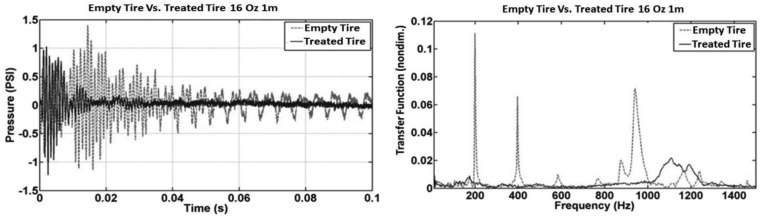
Time and frequency domain plot of the result of acoustically treated tire.

This test was conducted to validate the use of the foam insert to reduce resonance effect due to tire cavity. The test configuration of Figure 3 was used, along with a 1 lb. hammer strike approximately one meter distant. The results are the average of three hammer strikes. As is evident from the time domain result in the left of Figure 8, the DTPS with foam liner installed in the tire exhibited a significant reduce of signal damping compared to the case without the foam liner. A frequency analysis of the DTPS and directional microphone in the right of Figure 6 reveals that the resonant response of the tire system can be significantly improved through the use of acoustic damping foam. Signal to noise is significantly increased in the 900–1600 Hz range as compared to the case without such material.

### 4.3. DTPS Representing/Reconstructing Ground Acceleration

**Figure 9.**
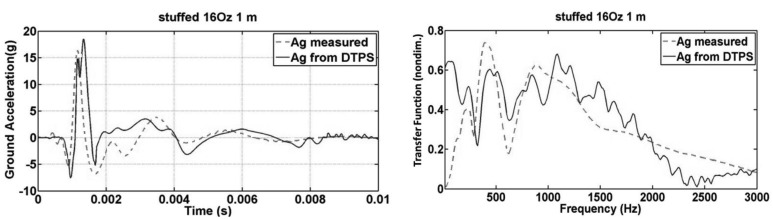
Time and frequency plots of reconstructed ground acceleration Ag (Ag from DTPS) and measured ground acceleration (*A_g_* measured) of 1 lb. hammer impact.

The test configuration of Figure 3 was used, along with ¼ lb., 1 lb. and 4 lb. hammer strike approximately at a distance of one meter. The results are the average of three hammer strikes. Equation (3) is used for calculating/reconstructing ground acceleration. The result is comparable to the actual measured data from ground-mounted accelerometer as shown in Figure 9 for both time domain and frequency domain. With the inserting of acoustic foam, and 190 Hz and its harmonic peaks was removed for better approximation of the accelerometer data.

**Figure 10.**
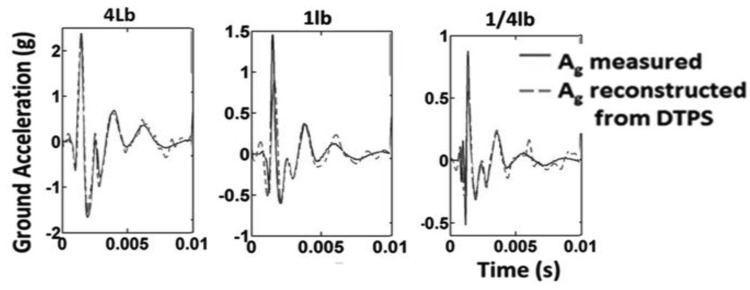
Time plots of reconstructed ground acceleration of 4 lb., 1 lb., and ¼ lb. hammer impacts.

Other results of different hammer impacts are shown in Figure 10. A cubic spline was used on the data from the plural impacts after applying the calculation for reconstructing ground acceleration. A very good prediction of the actual ground acceleration is achieved. Since the most useful information is found within 5 milliseconds of the ground acceleration signal. Since the acoustic radiation from the surface wave is significantly stronger than acoustic noise, subsurface information may be derived without the need for a ground-mounted accelerometer.

### 4.4. Further Tests of DTPS versus Ground Vibration/Acceleration

**Figure 11.**
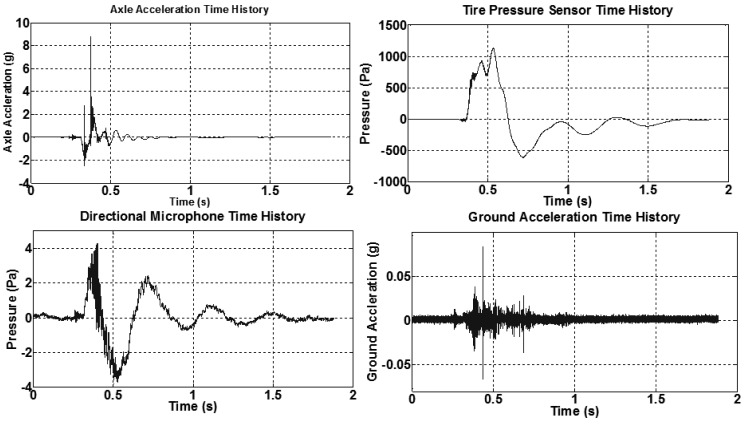
Results of the four sensors of low frequency vehicle impact test.

**Figure 12.**
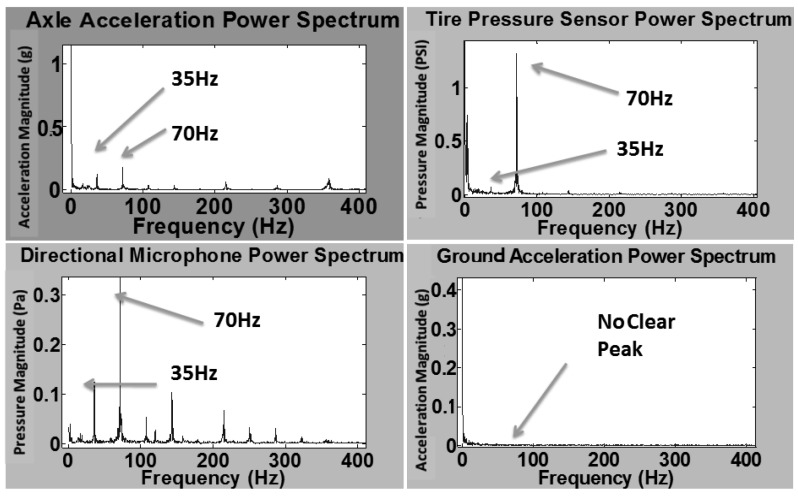
Results of the four sensors of high frequency vehicle impact test.

Another test of the same setup described in Section 3.2 was also performed, but the input source was provided by the tire instead of a hammer. This is achieved by jacking up car and releasing (low frequency impact) it and also by putting a generator (Honda EU2000i) inside the test van closer to the test tire (high frequency impact). Figure 11 shows results of four sensors in both time and frequency domains of the low frequency vehicle input, while Figure 12 illustrates results of the high frequency vehicle input.

In Figure 11, transfer functions of different sensors are calculated as follows: (1) average transfer ratio *T_1_* = *A_ground_/A_axle_* = 0.002; (2) average transfer ratio *T_2_* = *P_tire_/A_axle_* = 180 (Pa/g), where *A_ground_* is the ground acceleration, *A_axle_* is the axle acceleration and *P_tire_* is the dynamic pressure change. In Figure 12, same frequency peaks at 35 Hz and 70 Hz are shown for all three sensors except the ground accelerometer. This is most likely related to the lower impact amplitude of the high frequency impact. Transfer functions of different sensors are calculated as follows: (1) average transfer ratio *T_1_* = *A_ground_/A_axle_* = 0.001; (2) average transfer ratio *T_2_* = *P_tire_/A_axle_* = 1000 (Pa/g); (3) average transfer ratio *T_3_* = *P_tire_/P_dir_* = 3, where *P_dir_* is pressure of the directional microphone. From the results of transfer function of these two tests, low *T_1_* indicates that axle acceleration doesn't excite much ground vibration, which is less than 0.2%. Moreover, high values of *T_2_* and *T_3_* confirm that the DTPS responds directly to axle vibration without responding to large amounts of ambient noise.

## 4. Conclusions

In summary, an instantaneous, lightweight, and economical DTPS system has been designed and evaluated with hardware components and associated supporting signal processing strategies for measurement of ground vibration. The DTPS offers a sensing option that is easy to install on existing valve stems. It has been shown that the tire pressure changes are dominated by ground vibration and that the DTPS amplified ground vibration in its operating frequency range, especially in the frequency range of 0–3,000 Hz. Moreover, acoustic damping treatment insert inside the tire reduced the resonant response the tire cavity, which yielded higher SNR. Ground acceleration derived from the DTPS measurement was a close match to the ground acceleration measured by ground-mounted accelerometers, which indicates the potential of the DTPS to measure a surface wave generated by a tire while the vehicle is moving.

### Limitations

This work is focused on the development of a method for ground acceleration testing of pavements. Extensive evaluation of the method under many different conditions remains to be conducted. Although more than 50 data sets have been collected during the development, only a few detailed case studies are presented in this work. The presented technique is based solely on simplified tire system under adiabatic condition with one DTPS measurements without analysis of system properties for various temperatures. A calibration of transfer function between dynamic tire pressure change and ground acceleration may be needed for different tire system or for more accurate application.
